# Valorization of Wild-Type *Cannabis indica* by Supercritical CO_2_ Extraction and Insights into the Utilization of Raffinate Biomass

**DOI:** 10.3390/molecules28010207

**Published:** 2022-12-26

**Authors:** Falguni Pattnaik, Nidhi Hans, Biswa R. Patra, Sonil Nanda, Vivek Kumar, Satya Narayan Naik, Ajay K. Dalai

**Affiliations:** 1 Centre for Rural Development and Technology, Indian Institute of Technology Delhi, New Delhi 110016, India; 2 Department of Chemical and Biological Engineering, University of Saskatchewan, Saskatoon, SK S7N 5A9, Canada

**Keywords:** supercritical CO_2_ extraction, *Cannabis indica*, cannabinoids, raffinate biomass, antioxidants

## Abstract

Supercritical CO_2_ extraction (SCCO_2_) extraction of cannabis oil from Indian cannabis (*Cannabis indica*) leaves was optimized through a central composite design using CO_2_ pressure (150–250 bar), temperature (30–50 °C) and time (1–2 h). From the regression model, the optimal CO_2_ pressure, extraction temperature and time were 250 bar, 43 °C and 1.7 h, respectively resulting in the experimental yield of 4.9 wt% of cannabis oil via SCCO_2_ extraction. The extract contained cannabidiol, tetrahydrocannabivarin, Δ^9^-tetrahydrocannabinol and Δ^8^-tetrahydrocannabinol as well as two terpenoids such as cis-caryophyllene and α-humulene. Besides SCCO_2_ extraction of cannabis oil, the raffinate biomass was utilized to extract polyphenols using water as the extraction medium. Cannabis oil and water extractive were investigated for their half-maximal inhibitory concentration (IC_50_) values, which were found to be 1.3 and 0.6 mg/mL, respectively. This is comparable to the commercially available antioxidant such as butylated hydroxytoluene with an IC_50_ value of 0.5 mg/mL. This work on SCCO_2_ extraction of cannabinoids and other valuable bioactive compounds provides an environmentally sustainable technique to valorize cannabis leaves.

## 1. Introduction

*Cannabis indica* is an herbaceous species belonging to the family of Cannabaceae and is widely recognized because of its unparalleled versatility in medicinal and therapeutical properties. Cannabis plants are of tremendous interest due to the presence of secondary metabolites such as cannabinoids, flavonoids, terpenoids, alkaloids, lignans, anthocyanins, and quinones in their leaves and female flowers [1]. Among these various bioactive compounds, cannabinoids are the most abundant constituents of cannabis flowers and leaves. Although the female flower or bud (also considered secondary leaves) mainly constitutes cannabinoids, the cannabis leaves also contain a considerable content of cannabinoids. Cannabis leaves, buds and flowers contain more than hundreds of cannabinoids, the content of which varies within species and strains [2]. The variation in the contents of different cannabinoids is also dependent upon climatic and other growth conditions of the cannabis plants [3].

The main psychoactive component of cannabis is tetrahydrocannabinol (THC). However, cannabis plants contain more than 400 bioactive components such as cannabinoids, terpenes, fatty acids, flavonoids, essential oils and polyphenols [3]. Cannabidiol (CBD) is another non-psychoactive cannabinoid compound found in high concentrations in cannabis, which has shown a high obstructing effect on the impact of THC on the nervous system [4]. Cannabis has potential applications in the treatment of various conditions such as Alzheimer’s disease, glaucoma, cancer, anxiety, neuropathic pain and depression. Furthermore, cannabis extracts have been utilized in the alleviation of side effects resulting from cancer treatment and in the therapy of patients suffering from acute immunodeficiency syndrome (AIDS) [5]. Unlike THC, CBD is not a psychotropic cannabinoid that influences the central nervous system and specific areas of the brain. However, its affinity for the serotonin system is intriguing as it explains the anti-depressant and stress-relieving properties [3,6]. Moreover, due to the non-psychoactive nature of CBD, it is well-popularized among consumers to cure various diseases with no or very few side effects.

As per usage, cannabis is classified into drug type and non-drug type. The drug type is usually rich in cannabinoids, while the latter is mainly fiber-type cannabis containing insufficient cannabinoids amount. Although cannabis has valuable medicinal properties still it is an untapped bioresource as its legal usage is controlled in various parts of the world [3]. The sole reason behind this is substance abuse, which can lead to psychosis, mental disorders and consequential anti-social incidents. Interestingly, cannabis infusion in food and beverage products has evolved into one of the biggest markets after its legalization in some countries [7]. The increase in demand for cannabis extracts in medical and food products is unprecedented. However, the extraction of cannabis extracts is challenging. Cannabis oil is generally bitter in taste and highly viscous [8]. Therefore, its extraction method and technology are highly important for ensuring purity, quality and quantity.

Bioactive components from cannabis biomass can be extracted using various extraction processes and alcohol-based solvents. The quality of the final extraction products is highly influenced by the extraction method. The concentration of THC and other cannabinoids is also process-specific. The use of solvents can pose safety concerns related to their flammability and toxicity for human consumption. Among all the technologies, supercritical solvent extraction technology is considered one of the most efficient processes for the extraction of essential oils and secondary metabolites (bioactive compounds) from different herbs and vegetable matrices [5,9]. The microwave-assisted extraction process has also been reported to extract proactive antioxidant compounds from biomass [10]. The most favorable solvent used in supercritical extraction is carbon dioxide (CO_2_) because of its low cost, abundance and valorization potential. Moreover, CO_2_ attains a supercritical state at room temperatures (31.1 °C) and slightly higher pressure (74 bar). The main advantage of using CO_2_ as a solvent is that the final product is free from solvent, which eliminates the use of extra downstream processes for solvent separation. The bioactive compounds present in cannabis are temperature and solvent-sensitive. Hence, supercritical CO_2_ (SCCO_2_) extraction is operated at ambient conditions to favor the extraction of cannabinoids without compromising their properties. The low polarity of SCCO_2_ is the only concern, which can be overcome effectively by employing polar modifiers such as water and alcohol [11]. CO_2_ is considered a non-polar molecule due to the linear bonding structure and the target compounds such as cannabinoids possess comparatively more polarity due to the presence of phenolic groups and carboxylic groups [12]. However, in SCCO_2_, high CO_2_ pressure results in the deformation of CO_2_ bonds, which subsequently increases its polarity synchronizing with that of the cannabinoids [13].

There is rare literature available on the SCCO_2_ processing of cannabis biomass to extract bioactive compounds. Rovetto and Aieta [11] investigated different CO_2_ pressures, e.g., 170, 240 and 340 bar at a constant temperature of 55 °C and ethanol as the co-solvent for the extraction of cannabinoids from the buds (or flowers) of different nursery-grown *Cannabis sativa* plants. Maximum yields of cannabinoids (8–19 wt%) were obtained at the highest CO_2_ pressure of 340 bar. Kitrytė et al. [14] also studied SCCO_2_ extraction of cannabinoids from the *C. sativa* flowers and leaves. Naz et al. [15] studied the optimization of the SCCO_2_ process for the extraction of different terpenoids from *Cannabis* plants using different pressures (80, 85 and 90 bar) and temperatures (40, 45 and 50 °C). In a recent study, Jokić et al. [16] optimized the SCCO_2_ extraction of terpenoids and cannabinoids from *C. sativa* flowers to obtain a cannabis oil yield of 8.8 wt% at CO_2_ pressure of 320 bar and extraction temperature of 40 °C.

All the above-mentioned studies focused on SCCO_2_ extraction of terpenoids and cannabinoids from cannabis flowers grown under controlled and regulated conditions. However, these studies do not provide much information on the usage of the raffinate cannabis biomass after the extraction process. In addition, there are no studies regarding the utilization of wild-type cannabis leaves. In this study, wild-type *C. indica* leaves and raffinate cannabis biomass were used as the feedstock for SCCO_2_ extraction to recover cannabinoids and high-value terpenoids using statistical modeling and process optimization. Crude extract obtained from SCCO_2_ extraction of cannabis leaves was subjected to deep freezing and column chromatography to separate wax and residual chlorophyll, respectively. The raffinate biomass obtained from SCCO_2_ extraction was used as the substrate for extracting other industrially relevant bioactive compounds using the conventional solvent extraction using hexane, ethanol and water as the media. The cannabinoid mixture (cannabis oil) obtained from the optimized conditions and extractives generated from the water extraction were characterized using spectroscopy, chromatography and mass spectrometry to determine their composition, antioxidant behavior and biochemical profiles.

## 2. Results and Discussion

### 2.1. Supercritical CO_2_ Extraction of Cannabis Oil

SCCO_2_ extraction of cannabis oil from *Cannabis indica* leaves was conducted through the central composite design (CCD) design of experiments by assessing three experimental factors such as CO_2_ pressure, temperature and extraction time with cannabis oil yield as the response. The cannabis oil was obtained in the range of 2.2–4.9 wt% of the feedstock as presented in Table 1. The highest cannabis oil yield was obtained at 250 bar of CO_2_ pressure in 2 h at 30 °C, whereas the lowest cannabis oil was attained at the CO_2_ pressure of 150 bar in 1 h at 30 °C. Although increasing the extraction temperature increases the polarity of CO_2_ as well as the diffusibility of the medium, in this study, its effect was minimal for the oil yield [17]. The SCCO_2_ extraction time positively affected the cannabis oil yield. With the increase in the extraction time, cannabis oil yield also augmented up to 1.5 h and then declined due to the degradation of cannabinoids and/or higher polarity [13,18,19].

### 2.2. Statistical Assessment of SCCO_2_ Extraction Process through a Regression Model

The effects and interactions of various experimental parameters on cannabis oil yield were evaluated by observing the statistical factors obtained from the regression model. Eighteen sets of experiments were conducted, the results of which were confirmed in terms of the acceptability of the regression or statistical model by evaluating different coefficients. The Design-Expert program was used to optimize the process variables using the experimental model. To derive the regression equation, the findings from the experimental batches were fitted in several statistical models such as cubic, quadratic, linear and two-factor interaction. This created a link between cannabis oil yield and experimental variables (i.e., CO_2_ pressure, extraction time and temperature). The rationality of the regression model was determined using statistical properties such as the sequential model sum of squares and the summary of the model [20,21]. The adequacy of the generated regression model was confirmed using the sum of squares (Table 2) and summary statistics (Table 3). Table 4 shows that the acceptance of the statistical model was influenced by two factors, i.e., a high F-test value and a low probability value (*p*-value) [22,23]. Table 4 shows that the F-test value and *p*-value were 136 and <0.0001 for the regression model indicating that the model was acceptable. The model is significant if the *p*-value is <0.05 and the F-test value is of greater value. The lack of fit was another important consideration in determining the model’s suitability. The lack of fit *p*-value for this regression model was 0.63, indicating that the model was significant. It should be highlighted that the *p*-value for lack of fit should be more than 0.05, which was achieved for this statistical study.

The acceptability of the regression or statistical model depends on the R^2^ value (coefficient of determination), adjusted R^2^ and predicted R^2^. The validation of various terms in the model summary statistics (Table 3) have a major impact on the adequacy of the regression model, which is dependent on R^2^, adjusted R^2^ and predicted R^2^ [24,25]. According to Table 3, the quadratic model was well-fitted with the experimental results. The adjusted R^2^ and predicted R^2^ of 0.98 and 0.95, respectively were significantly related to each other. It was discovered that the difference between the two values was 0.03 (less than the permissible limit of 0.2). The *p*-value, F-value, and R^2^ values determined the regression model to be extremely significant. Following the determination of the significance of the regression model, a regression equation was derived, as indicated in Equation (1).

(1) $$\mathrm{Cannabis}\;\mathrm{oil}\;\left(\mathrm{wt}\%\right)=-4.74+0.01\;P+0.12\;T+3.35\;t-0.0005\;\mathrm{PT}-0.002\;\mathrm{Pt}+0.0065\;\mathrm{Tt}+0.00007{\;P}^{2}-0.003{\;T}^{2}-0.95\;t^{2}$$

where, P, T and t represent CO_2_ pressure (bar), temperature (°C) and extraction time (min), respectively. PT, Pt and Tt denote the interactions of CO_2_ pressure and temperature, CO_2_ pressure and time, and temperature and time, respectively. P^2^, T^2^ and t^2^ are the square terms for CO_2_ pressure, temperature and extraction time, respectively.

**Figure 1:**
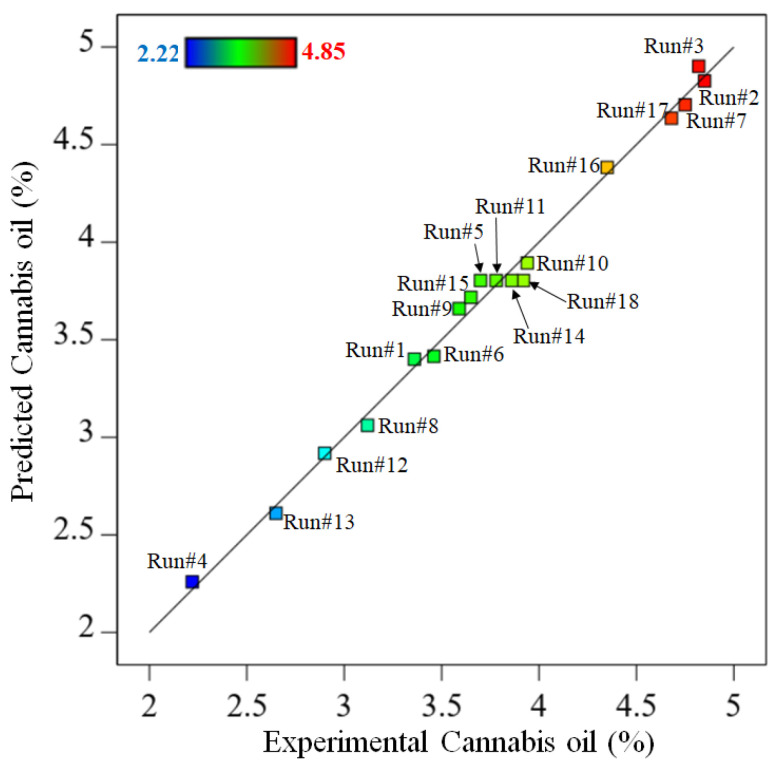
Parity plot between the experimental and predicted cannabis oil yield.

The present regression model had a higher F-value and a *p*-value < 0.05, indicating its acceptance. Furthermore, the lower *p*-value for each factor such as CO_2_ pressure, temperature and extraction time in the model (*p*-value < 0.05) reflects the credibility of the significance of the regression model [26]. The *p*-value for individual factors such as CO_2_ pressure (A), temperature (B), extraction time (C) and interactive factors such as AB, A^2^ and C^2^ were found to be less than 0.05. Hence, these regression factors were considered the prominent model variables. The parity plot (Figure 1) demonstrated a relationship between the experimental and predicted cannabis oil yields, which exhibited outstanding consistency across all model points. A trend in cannabis oil yield is depicted by a straight line in this figure. Except for a few experimental runs, the majority of the acquired data are aligned with or around the projected trend line. During certain experimental runs (e.g., #3, #5, #9, #14 and #15), the cannabis oil yield slightly differed from the expected values given by the model, which was due to the degradation of cannabinoids at higher temperatures or longer extraction times. The error (%) between the predicted and experimental yields of cannabis oil is presented in Table S1.

### 2.3. Effects of Different Parameters on Cannabis Oil Yield

A regression model depicts the influence of several experimental conditions (process parameters) on the response (cannabis oil yield). Furthermore, it elucidates the interaction of different experimental factors and their influence on the extraction process. The *p*-values of the factors, which should be less than 0.05, determine their importance for the extraction process. CO_2_ pressure (A), temperature (B), time (C), their squares (A^2^, B^2^ and C^2^), and the interaction between CO_2_ pressure and temperature (AB) showed *p*-values < 0.05 as presented in Table 4.

Figure S1 shows the effects of CO_2_ pressure on cannabis oil yield at a constant temperature and extraction time of 40 °C and 1.5 h, respectively. Cannabis oil yield increased from 3.1 wt% to 4.8 wt% with the rise in CO_2_ pressure from 150 bar to 250 bar. This was due to the increase in the polarity of SCCO_2_ as the extraction medium with the pressure increase [12]. Due to the increase in the polarity corresponding to the polarity of cannabinoids, the dissolution of these compounds increased resulting in a higher yield of cannabis oil. Although temperature also provided an equivalent trend, the effect of the temperature did not follow similar intensity as the effect of CO_2_ pressure.

Figure S2 represents the effects of the temperature of the extraction vessel on the yield of cannabis oil where the data points were presented between the range of 30–50 °C at a constant CO_2_ pressure and extraction time of 250 bar and 1.5 h, respectively. Although there was no significant effect of temperature on the cannabis oil yield, there was a slight decrease in the cannabis oil with the increase in the temperature from 30 °C to 50 °C. It was due to the increase in polarity of the SCCO_2_ with the increase in the deformation in the bond structure of the CO_2_ molecule, which dissolves more amount of more polar molecules than the cannabinoids [12,19]. Figure S3 presents the effect of extraction time (1–2 h) on the cannabis oil yield at the constant temperature of 40 °C and CO_2_ pressure of 250 bar. At these temperatures and CO_2_ pressure, the cannabis oil yield increased with time from 1 h to 1.5 h. However, with a further increase in the extraction time to 2 h, cannabis oil yield decreased. This was due to the release of volatile terpenes and other low-molecular weight compounds present in cannabis oil at prolonged extraction duration in the SCCO_2_ extraction process [12].

**Figure 2:**
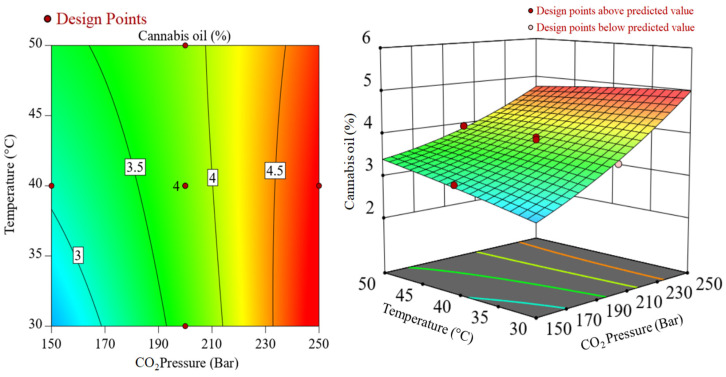
Interactions between CO_2_ pressure and temperature during SCCO_2_ extraction at a constant extraction time of 1.5 h.

**Figure 3:**
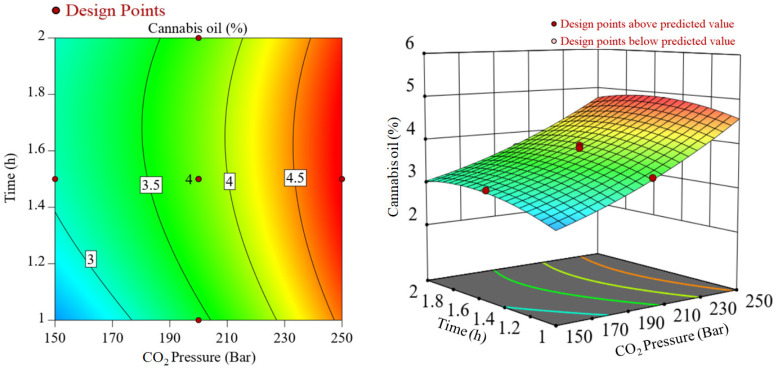
Interactions between CO_2_ pressure and time during SCCO_2_ extraction at a constant temperature of 40 °C.

The interaction effects of CO_2_ pressure and temperature during SCCO_2_ extraction of cannabis oil at a constant extraction time of 1.5 h are represented in Figure 2. In Figure 2, significant interactions between the two variables, i.e., temperature and CO_2_ pressure were noticed. While considering the lower CO_2_ pressure such as 150 bar, there was an increase in cannabis oil yield, whereas, under higher CO_2_ pressure of 250 bar, cannabis oil yield declined at higher temperatures. It may be due to the retrograde condensation behavior of SCCO_2_ at high pressures and temperatures and/or the removal of highly volatile terpenes under high CO_2_ pressures, e.g., 250 bar [27]. Similarly, Figure 3 shows the interactive plot between CO_2_ pressure and extraction time at a constant temperature of 40 °C on cannabis oil yield. Cannabis oil yield increased with rising CO_2_ pressure and temperature although pressure had a more prominent effect than temperature and extraction time. The interactions between the numerous components as well as the influence of single factors on the yield of cannabis oil are discussed after the regression model was verified using various parameters. The procedure culminated with the retrieval of the optimization conditions from the respective databases (Figure S4). The expected yield of cannabis oil was determined using the optimum process parameters. After implementing these optimized conditions, a run of optimization was performed to assess the cannabis oil yield with an experimental yield of 4.9 wt%. Equation (2) was used to calculate the experimental error, which was found to be 2.03 wt%.

(2) $$\mathrm{Percent}\;\mathrm{error}\;\left(\%\right)=\frac{\mathrm{Experimental}\;\mathrm{yield}\;\left(\mathrm{wt}\%\right)-\;\mathrm{Predicted}\;\mathrm{yield}\;\left(\mathrm{wt}\%\right)\;}{\mathrm{Experimental}\;\mathrm{yield}\;\left(\mathrm{wt}\%\right)}\times 100$$

### 2.4. Water Extraction of Raffinate Cannabis Leaves

After the SCCO_2_ extraction of cannabis leaves at the optimal conditions generated from the regression model (CO_2_ pressure: 250 bar, temperature: 43 °C and time: 1.7 h), the raffinate biomass (residual leaves) was treated with boiling water in a conventional solvent extraction process to extract polyphenols. The leafy residue constitutes residual cannabinoids, terpenoids, chlorophyll and a significant amount of polyphenols. The extraction of polyphenols can add value to the SCCO_2_ extraction of cannabis oil. Before the water extraction, the residual biomass obtained at the optimized SCCO_2_ extraction conditions was extracted with hexane and ethanol as solvents, with yields of 3.1 wt% and 12.3 wt%, respectively. These extractives majorly constitute chlorophyll, which was confirmed using UV-Visible spectroscopy. Although a major portion of the ethanolic extract is chlorophyll, the extract can contain some phenolic polar groups. This is attributed to the higher polarity of ethanol, which can leach out the polar compounds from the biomass matrix. However, highly polar compounds with greater numbers of phenolic groups cannot be extracted using ethanol due to their lower polarity than the desired polyphenolic compounds. The extraction of these highly polar polyphenols can be achieved by implementing water as the extraction medium. From the water extraction of raffinate cannabis leaves, the yield of water-soluble constituents was found to be 28.3 wt%.

Extractives obtained from the water contain various antioxidants such as polyphenolic compounds. The total phenolic content present in the water extractive was estimated to be 17.4 mg GAE/g of extract, whereas the total tannin content was found to be 5.6 mg GAE/g. The water extractive isolated from the raffinate cannabis leaves was dark brown resinous and sticky in texture, which may be due to the presence of polymeric compounds like tannins or other polyphenols. Although the hot water extraction of leaves through the Soxhlet apparatus possessed a higher extraction yield, the prolonged extraction time and higher boiling temperature (100 °C) can affect the effectiveness of the bioactive compounds due to the thermal degradation of the aromatic polyphenols [28].

### 2.5. Characterization of Cannabis Oil and Water Extractive

The cannabis oil obtained from SCCO_2_ extraction of cannabis leaves at the optimized conditions (i.e., CO_2_ pressure: 250 bar, temperature: 43 °C and time: 1.7 h) was characterized by gas chromatography-mass spectrometry (GC-MS) to determine the percentage of different constituents present in the extractive. The retention time, area and chemical structures of these constituents are presented in Table 5. Among these five main compounds identified from the GC-MS, CBD and THC (∆^8^ or ∆^9^ isomers) were the prominent portions in the cannabis extract constituting 29 wt% and 35 wt% (with respect to cannabis oil extract), respectively. Both these cannabinoids possess various medicinal activities for various disorders and medical conditions such as anxiety, insomnia, Parkinson’s disease, Huntington’s disease, Alzheimer’s disease as well as inflammatory bowel disease, post-Ebola syndrome, nausea and cancer [29,30,31,32,33].

Besides THC and CBD, another cannabinoid, tetrahydrocannabivarin (THCV) was also detected in the GC-MS characterization of cannabis extract constituting 8%. THCV is a THC homolog with a propyl side chain rather than a pentyl side chain, as is the case with THC [3]. Besides, the cannabinoids, two isomeric terpene molecules such as cis-caryophyllene and α-humulene were also detected in the extract with the composition of 5% and 3%, respectively. These terpenes have various therapeutic activities for depression, insomnia, anxiety, digestive disorders and convulsions. Other terpenes such as linalool, myrcene, limonene, ocimene, pinene and terpinolene are also found in various strains of cannabis. From Table 5, the retention time of cis-caryophyllene and α-humulene was less due to the presence of aliphatic groups in the cannabinoid molecules [11]. Similarly, the retention time of CBD and THC were higher than the THCV, because of the presence of a larger number of carbon atoms in the aliphatic groups. The cannabis extract obtained from SCCO_2_ extraction contained a comparatively lesser number of constituents, which can decrease the complexity of the extract and also subsequently reduce the cost of the downstream processes for isolating the constituents.

**Figure 4:**
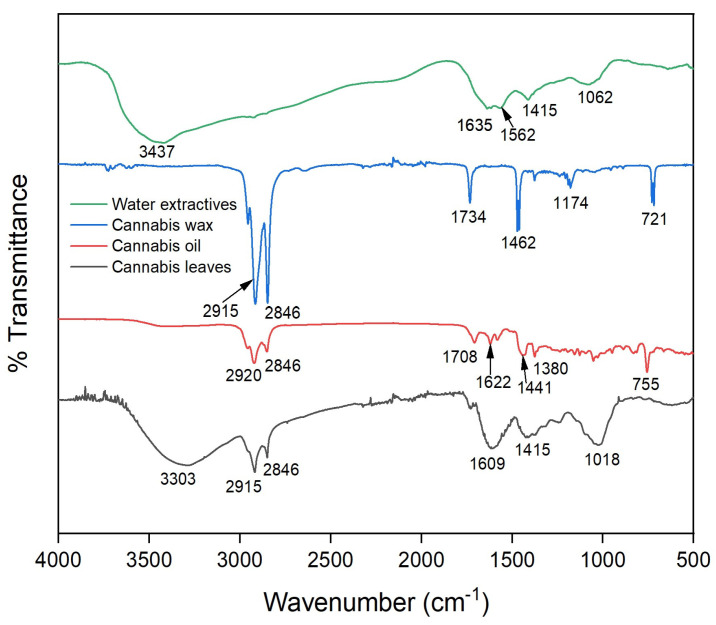
FTIR spectra of cannabis leaves, cannabis oil and wax obtained from SCCO_2_ extraction at optimized conditions (CO_2_ pressure: 250 bar, temperature: 43 °C and time: 1.7 h).

Figure 4 presents the Fourier-transform infrared (FTIR) spectra of cannabis leaves, cannabis oil, cannabis wax and water extractive. The FTIR peak assignments of these bands are presented in Table 6. The broad peaks at 3303–3437 cm^−1^ for cannabis leaves, cannabis oil and water extractive were assigned to alcohols and phenols having hydroxyl groups [34]. However, this broad peak was present in the case of cannabis wax due to the absence of the hydroxyl group indicating its hydrophobicity. All the samples except the water extractive had two sharp peaks (more prominent for the cannabis wax) at 2800–2920 cm^−1^ assigned to the aliphatic groups [34]. The peaks at 1700–1750 cm^−1^ in cannabis leaves and cannabis wax were due to the C=O group of ester or any other carbonyl groups. Similarly, the peak in the range of 1600–1650 cm^−1^ was designated to aromatic C=C linkages, which was absent in the case of cannabis wax [35].

**Figure 5:**
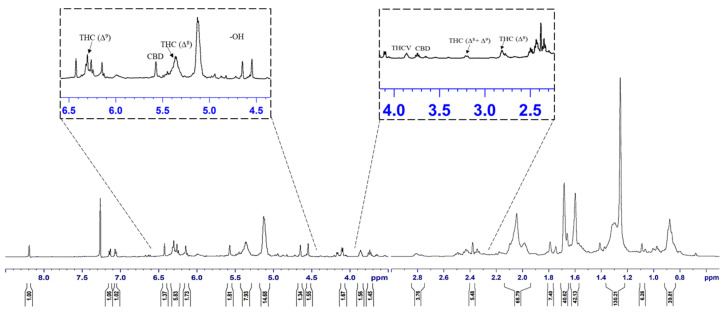
^1^H NMR spectra of cannabis oil extracted using SCCO_2_.

Figure 5 represents the nuclear magnetic resonance (^1^H NMR) spectra of cannabis oil where the peaks at 0.85, 1.25, 1.6, 1.7, 2.1, 2.8, 3.2, 3.75 and 3.85 ppm represented the aliphatic or alkane-based protons present in –CH, –CH_2_ and –CH_3_ groups in Δ^8^-THC, Δ^9^-THC, CBD and THCV. Among these cannabinoids, Δ^8^-THC, Δ^9^-THC and THCV possess similar structural features except for two lesser carbon atoms in the aliphatic side chain of THCV [31]. Therefore, for the higher chemical shifts (δ_H_ > 4 ppm), similar peaks were assigned to THC and THCV. Considering the structural feature of CBD, unlike THC and derivatives, CBD has an alkene-based = CH_2_ group (vinylic) at position 9 (Figure S5a). The prominent peak at 5.1 ppm represented the vinylic proton present at position 9 of CBD [36]. The group of peaks between the chemical shift of 4.5–5 ppm represents the phenolic proton of –OH groups present in the cannabinoids, especially THC. The –CH proton present at position 5 (Figure S5b–d) of THC and THCV were represented by the peak at the chemical shift of 6.15 ppm. The position 6 (Figure S5b–d) protons of aromatic –CH groups of THC and THCV were represented by the peak at 6.3 ppm [36]. The detailed assignments for the different peaks are summarized in Table 7, whereas different proton positions are marked on the structures of concerned cannabinoids such as Δ^8^-THC, Δ^9^-THC, THCV and CBD.

At ambient temperatures, 2,2-diphenyl-1-picrylhydrazyl (DPPH) is a stable free radical that, when combined with an electron or hydrogen radical, forms a stable molecule. The potential of the DPPH radical to undergo reduction was determined by measuring the decrease in its absorbance at 517 nm. The reaction between the antioxidant molecule and the radical causes the radical to be scavenged by hydrogen donation, thus resulting in a drop in DPPH absorbance at 517 nm. At various concentrations, cannabis oil and water extractive had antioxidant activity comparable to normal butylated hydroxytoluene (BHT). At a dosage of 4 mg/mL, the percentage inhibition by cannabis oil and water extractive was 90 wt% and 81 wt%, respectively.

**Figure 6:**
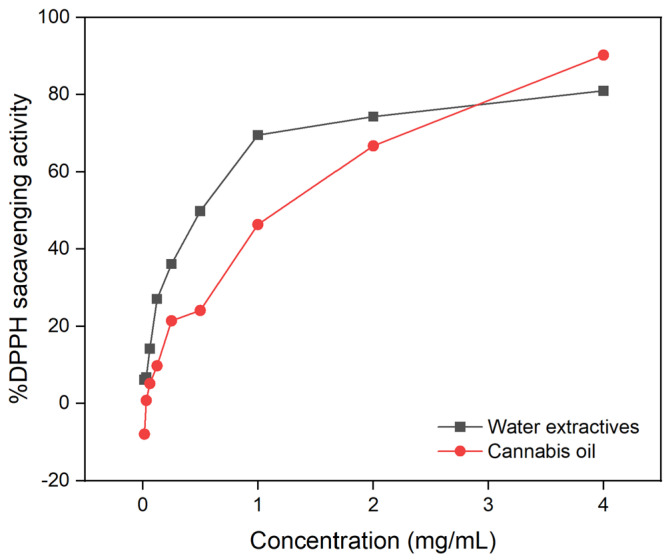
DPPH scavenging activity of water extractive and cannabis oil.

Figure 6 shows a comparison of the DPPH radical scavenging activity of water extractive and cannabis oil. In comparison to cannabis oil, water extractive had the most efficient DPPH radical scavenging potency. Water extractive had a half-maximal inhibitory concentration (IC_50_) value of 0.6 mg/mL compared to that of cannabis oil (1.3 mg/mL). Water extractive exhibited significant DPPH radical scavenging, which could be related to the presence of polyphenolic compounds in the extract. In comparison to oil and water extractive, BHT had a stronger radical scavenging activity (IC_50_ of 0.5 mg/mL).

## 3. Materials and Methods

### 3.1. Materials

**Figure 7:**
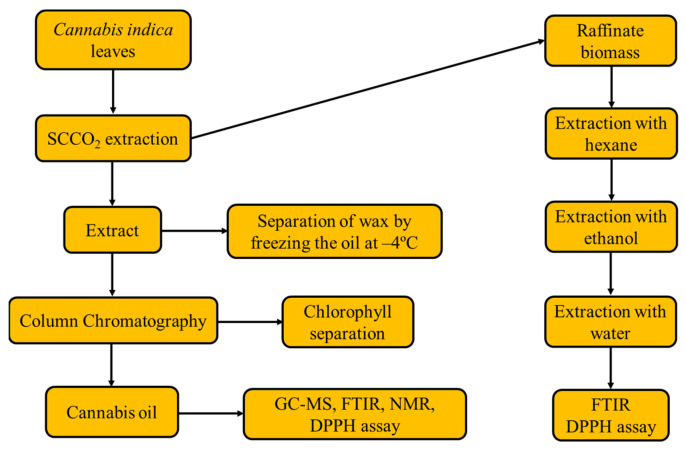
Process flow diagram of experiments and analyses in this study.

*C. indica* leaves were collected from the Haridwar region (Gaindi Khata Village Cluster) of Uttarakhand, India. The leaves were plucked from cannabis plants, air-dried and pulverized to obtain a particle size of 0.5 mm. Solvents such as hexane, ethanol, deuterated chloroform and DPPH having purities of ≥99% were purchased from Sigma Aldrich–Merck (Bengaluru, Karnataka, India). The experimental flow diagram is shown in Figure 7.

### 3.2. Supercritical CO_2_ Extraction of Cannabis Oil

SCCO_2_ extraction of cannabis oil was optimized using experiments through CCD where CO_2_ pressure, temperature and time were taken as the process parameters, whereas cannabis oil yield was taken as the response of the regression model. A total of 18 extraction sets were deduced from the statistical design using Design-Expert v11 (Stat-Ease, Minneapolis, MN, USA), which included four center points. The statistical model was designed for the extraction process by taking the above parameters where CO_2_ pressure, temperature and time were varied as 150–250 bar, 30–50 °C and 1–2 h, respectively. The designed experimental sets were presented in Table S2.

SCCO_2_ extraction was performed using a Waters Supercritical CO_2_ Extraction System (Waters Corporation, Milford, MA, USA). Before starting the extraction process, 100 g of pulverized and dried cannabis leaves were filled into the extraction vessel. CO_2_ gas was supplied to the extraction vessel by passing it through a heat exchanger with a constant flow rate of 35 g/min. The required time to achieve the CO_2_ pressure was varied between 10–20 min as per the test temperature and pressure. The operating CO_2_ pressure of the extraction process was controlled by an automatic back pressure regulator. After the extraction time, CO_2_ pressure inside the extraction vessel was gradually decreased to ambient pressure. The extractives were collected from the collection vessel. The extractives stuck to the wall of the collection vessel were washed with hexane and processed to separate chlorophyll, waxes and other impurities. The resinous extract obtained from the extraction process was collected from the vessel by dissolving it in ethanol and evaporating it using a rotary evaporator. The crude extract obtained from this extraction was termed the total extractive yield, from which wax was isolated through deep freezing, whereas residual chlorophyll was separated using column chromatography. The final wax and chlorophyll-free extract obtained was termed cannabis oil.

### 3.3. Solvent Extraction of Raffinate Biomass Obtained from SCCO_2_ Extraction

The raffinate biomass (residual cannabis leaves) obtained from SCCO_2_ extraction at the optimized conditions (CO_2_ pressure: 250 bar, temperature: 43 °C and time: 1.7 h) was used as the feedstock for the water extraction to recover polyphenolic compounds. Before the water extraction, various residual non-polar compounds (e.g., fatty acids) and polar compounds (e.g., phenolics and chlorophyll) were extracted using sequential solvent extraction using hexane and ethanol for 6 h and 8 h, respectively. After the extraction, the same feedstock was loaded into the Soxhlet extraction apparatus to recover highly polar polyphenols using water as the solvent. Water extraction of the feedstock was performed for 12 h. After the completion of each set of extraction, the solvent was evaporated, and the extract was weighed. The water extractive obtained from the Soxhlet extraction was analyzed by the method described in Tambe and Bhamber [37] to estimate the total phenolic content and tannin.

### 3.4. Characterization of Cannabis Oil and Cannabis Extracts

The cannabis extract obtained from the optimized conditions was analyzed using GC-MS to determine its organic composition. The GC-MS analysis was performed using a PerkinElmer Clarus SQ8 GC-MS system (PerkinElmer India, Maharashtra, India) equipped with an Elite-5ms capillary column (30 m × 0.25 mm × 0.25 µm). The oven temperature was kept at 120 °C for 1 min followed by an increase to 295 °C for 13 min. The injector temperatures and volume were 295 °C and 1 µL, respectively. Some additional parameters such as helium carrier flow rate (1 mL/min), split ratio (1:100), electron ionization (70 eV) and mass-to-charge ratio (50–500 amu), scan time (0.8 s) and inter-scan delay (0.01 s) were also followed during the GC-MS analysis. The cannabinoid constituents present in the cannabis extract were identified by using the mass spectra matched with the Wiley library.

FTIR spectra of cannabis leaves, cannabis oil, cannabis wax and water extractive of raffinate cannabis leaves were recorded using Nicolet iS50 FTIR spectrophotometer (Thermo Fisher Scientific, Waltham, MA, USA) using the wavenumber of range 500–4000 cm^−1^. The spectral resolution of all the analyses was kept constant at 8 cm^−1^. A Bruker Avance III 500 NMR spectrophotometer (Bruker, Billerica, MA, USA) was used to record the ^1^H NMR spectrum of the recovered cannabis oil at a frequency of 500 MHz.

The scavenging effect on DPPH free radicals was measured using the procedures described by Blois [18] with a few modifications to determine the antioxidant activity of cannabis oil and water extractive. The organic solvents hexane and water were used to extract crude metabolites from cannabis biomass. A rotary vacuum evaporator was used to concentrate the extracts. Each extract was serially diluted with at least 5–6 dilutions. Each dilution aliquot (100 µL) was added to 100 µL of 0.1 mM DPPH (prepared fresh in methanol). The mixture was incubated for 30 min at room temperature in the dark. Using a Shimadzu UV-2700i UV-Visible spectrophotometer (Shimadzu Corporation, Kyoto, Japan) and methanol as a blank sample, the absorbance of the cannabis samples was measured at 517 nm. The ability of DPPH to scavenge free radicals was determined using Equation (3):

(3) $$\mathrm{DPPH}\;\mathrm{Inhibition}\;\mathrm{activity}\;\left(\%\right)\;=\frac{{\mathrm{Absorbance}}_{\mathrm{Control}}-\left({\mathrm{Absorbance}}_{\mathrm{Sample}\;+\;\mathrm{DPPH}}-{\;\mathrm{Absorbance}}_{\mathrm{Sample}}\right)}{{\mathrm{Absorbance}}_{\mathrm{Control}}}\times 100$$

By correlating the sample concentration and DPPH inhibition activity, the effective concentration of the sample required to scavenge DPPH by 50%, i.e., IC_50_ was calculated. The antioxidant activity of the extract was then compared to that of BHT as a control.

## 4. Conclusions

SCCO_2_ extraction can be considered one of the greener and more efficient extraction techniques for the recovery of cannabinoids from cannabis leaves. By taking CO_2_ pressure, temperature and extraction time as the process parameters, the extraction process was conducted through statistical modeling using a complex composite design of experiments. After process optimization, the statistical model was established as a quadratic model. CO_2_ pressure affected the extraction process more prominently than the other two parameters. The highest yield of cannabis oil (4.9 wt%) was obtained under the optimal SCCO_2_ conditions (e.g., CO_2_ pressure of 250 bar, temperature of 30 °C and extraction time of 2 h). However, the regression model generated the optimized CO_2_ pressure, temperature and time for SCCO_2_ extraction as 250 bar, 43 °C and 1.7 h, respectively with a theoretical yield of cannabis oil as 4.8 wt%. From FTIR, NMR and GC-MS, the cannabis oil extracted using SCCO_2_ was found to contain four major cannabinoids such as CBD, THCV, ∆^8^-THC and ∆^9^-THC including two characteristic terpenoids (e.g., cis-caryophyllene and α-humulene). The raffinate cannabis leaves obtained from SCCO_2_ extraction were used as the feedstock for recovering polyphenols using water extraction. The DPPH assay or antioxidant activity of the cannabis oil and water extractive determined their IC_50_ values as 1.3 mg/mL and 0.6 mg/mL, respectively. This fundamental study establishes SCCO_2_ as an environmentally friendly technique to extract various pharmaceutically relevant bioactive compounds from cannabis plants.

## Figures and Tables

**Table 1:** Yields of extractives from SCCO_2_ extraction of cannabis leaves.

Experimental Run	Process Parameters			Extractives Yield	
	CO_2_ Pressure (Bar)	Temperature (°C)	Time (h)	Total Yield (%)	Cannabis Oil (%)
1	150	50	2	5.9	3.4
2	250	30	2	7.3	4.9
3	250	40	1.5	7.1	4.8
4	150	30	1	3.6	2.2
5	200	40	1.5	6.1	3.7
6	200	40	1	5.6	3.5
7	250	30	1	7.2	4.8
8	150	40	1.5	5.4	3.1
9	200	30	1.5	5.9	3.6
10	200	50	1.5	6.2	3.9
11	200	40	1.5	6.1	3.8
12	150	50	1	3.5	2.9
13	150	30	2	5.8	2.7
14	200	40	1.5	6.2	3.9
15	200	40	2	6.1	3.7
16	250	50	1	6.7	4.4
17	250	50	2	7.3	4.7
18	200	40	1.5	6.2	3.9

Note: The data presented is an average of replicate measurements with a standard error of <3%.

**Table 2:** Model sum of squares for SCCO_2_ extraction of cannabis leaves.

Source	Sum of Squares	Degrees of Freedom	Mean Square	F-Value	*p*-Value	Remarks
Mean versus Total	253.88	1	253.88	-	-	-
Linear versus Mean	8.83	3	2.94	53.30	<0.0001	
2FI versus Linear	0.51	3	0.17	7.32	0.0057	
Quadratic versus 2FI	0.19	3	0.06	8.36	0.0076	Suggested
Cubic versus Quadratic	0.03	4	0.0084	1.16	0.44	Aliased
Residual	0.03	4	0.0072	-	-	-
Total	263.48	18	14.64	-	-	-

**Table 3:** Model summary statistics for SCCO_2_ extraction of cannabis leaves.

Source	Sequential *p*-Value	Lack of Fit *p*-Value	Adjusted R^2^	Predicted R^2^	Remarks
Linear	<0.0001	0.06	0.90	0.83	-
2FI	0.006	0.19	0.96	0.94	-
Quadratic	0.008	0.63	0.98	0.95	Suggested
Cubic	0.44	0.72	0.98	0.80	Aliased

**Table 4:** Analysis of variance (ANOVA) of the statistical model for SCCO_2_ extraction of cannabis leaves.

Source	Sum of Squares	Degrees of Freedom	Mean Square	F-Value	*p*-Value	Remarks
Model	9.54	9	1.06	136	<0.0001	Significant
CO_2_ pressure (A)	8.46	1	8.46	1085.63	<0.0001	-
Temperature (B)	0.14	1	0.14	17.56	0.003	-
Extraction time (C)	0.23	1	0.23	29.25	0.0006	-
AB	0.48	1	0.48	61.59	<0.0001	-
AC	0.03	1	0.03	3.39	0.10	-
BC	0.09	1	0.008	1.08	0.33	-
A^2^	0.08	1	0.08	11	0.01	-
B^2^	0.002	1	0.002	0.25	0.63	-
C^2^	0.15	1	0.15	19.55	0.002	-
Residual	0.06	8	0.008			-
Lack of fit	0.03	5	0.007	0.7608	0.63	Not significant
Pure error	0.03	3	0.009			-
Corrected total	9.60	17				-

**Table 5:** Main components identified by GC-MS of cannabis oil obtained from SCCO_2_ extraction at optimized conditions (CO_2_ pressure: 250 bar, temperature: 43 °C and time: 1.7 h).

Retention Time (min)	Area Percentage (%)	Molecules Detected	Molecular Structure
22.2	5	*cis*-caryophyllene	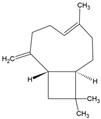
23.6	3	α-humulene	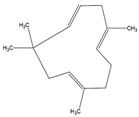
51.8	8	∆^9^-THCV	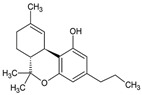
54.5	29	CBD	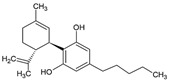
57.0	35	∆^9^-THC and ∆^8^-THC	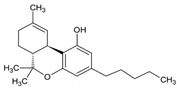 ∆^9^-THC 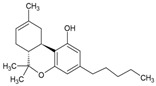 ∆^8^-THC

**Table 6:** Peak assignments for FTIR spectra of cannabis leaves, oil, wax and water extractive.

Cannabis Leaves	Cannabis Oil	Cannabis Wax	Water Extractive
1018 cm^−1^: C–O–C linkages of cannabinoids 1415 and 1609 cm^−1^: Aromatic –C=C– groups 1708 cm^−1^: C=O linkages of various groups or aromatics 2846 cm^−1^: Aliphatic C–H group (–CH_3_) 2915 cm^−1^: Aliphatic C–H group (–CH_2_) 3303 cm^−1^: –OH groups of phenols and moisture	755 cm^−1^: C–H linkages 1380, 2846 and 2920 cm^−1^: Aliphatic C–H groups 1441 and 1622 cm^−1^: Aromatic –C=C– groups 1708 cm^−1^: Aromatic rings 3400 cm^−1^: –OH groups of phenols and moisture	721, 1462 and 2915 cm^−1^: –CH_2_ groups 1174 cm^−1^: C–O linkages in ester groups 1734 cm^−1^: C=O group 2846 cm^−1^: –CH_3_ group	1062 cm^−1^: C–O linkages of polyphenols 1415 and 1635 cm^−1^: Aromatic –C=C– linkage 1562 cm^−1^: Phenol rings 3437 cm^−1^: –OH groups of phenols and moisture

**Table 7:** Peak assignments for ^1^H NMR spectrum of cannabis oil obtained from SCCO_2_ extraction.

Chemical Shift (δ_H_, ppm)	Assignments
0.85	Aliphatic –CH_3_ (position 5) of CBD, position 5 of Δ^8^-THC or Δ^9^-THC and position 3 of THCV
1.25	Aliphatic –CH_2_
1.6	–CH group
1.7	–CH group
2.1	Cyclic –CH_2_ group (non-aromatic)
2.8	Δ^8^-THC
3.2	Δ^8^-THC (–CH_2_; position 2) or Δ^9^-THC (–CH; position 2)
3.75	CBD (–CH; position 2)
3.85	THCV (–CH; position 3)
4.5–5	–OH group present in detected cannabinoids
5.1	=CH_2_ group of CBD
5.35	Δ^8^-THC
5.6	–CBD
6.15	Δ^8^-THC and Δ^9^-THC (–CH group; Position 5)
6.3	Δ^8^-THC and Δ^9^-THC (–CH group; Position 6)
7.25	CDCl_3_

## Data Availability

The data presented in this article are available on reasonable request from the corresponding author.

## Supplementary Materials

The following supporting information can be downloaded at: https://www.mdpi.com/article/10.3390/molecules28010207/s1, Figure S1: Effects of CO_2_ pressure on cannabis oil yield at constant temperature (40 °C) and extraction time (1.5 h); Figure S2: Effects of temperature on cannabis oil yield at constant CO_2_ pressure (200 bar) and extraction time (1.5 h); Figure S3: Effects of extraction time on cannabis oil yield at constant temperature (40 °C) and CO_2_ pressure (200 bar); Figure S4: Optimized conditions of SCCO_2_ generated from the statistical model; Figure S5: Chemical structures of (a) CBD, (b) Δ^9^-THC, (c) Δ^8^-THC and (d) THCV; Table S1. Comparison between the experimental and predicted yields of cannabis oil; Table S2. Design of experiments for SCCO_2_ extraction of cannabis leaves.
