# Supplementary material for: Valorization of Wild-Type Cannabis indica by Supercritical CO2 Extraction and Insights into the Utilization of Raffinate Biomass
Source: Molecules. 2022 Dec 26;28(1):207. doi: 10.3390/molecules28010207 (PMC9822091; doi:10.3390/molecules28010207)
Supplement: Supplementary file 1 [file molecules-28-00207-s001.zip › molecules-2087691-supplementary.pdf]

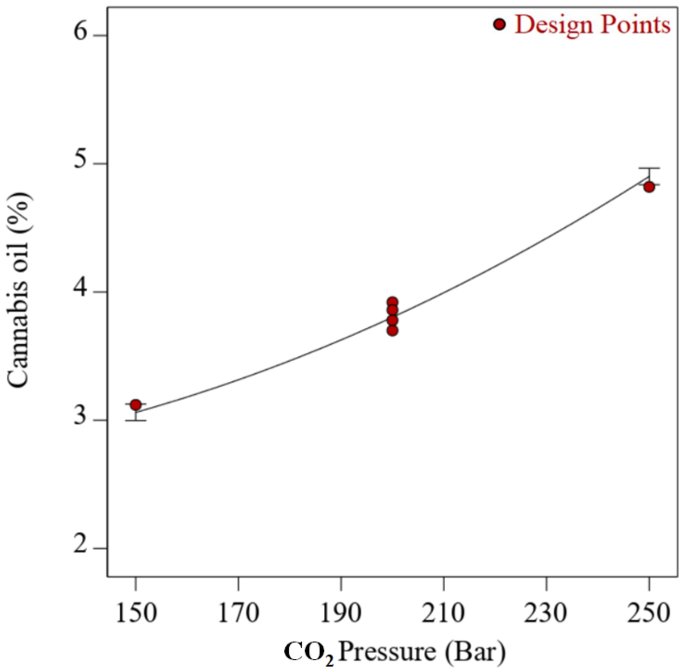

Fig. S1: Effects of CO_2 pressure on cannabis oil yield at a constant temperature (40°C) and extraction time (1.5 h)

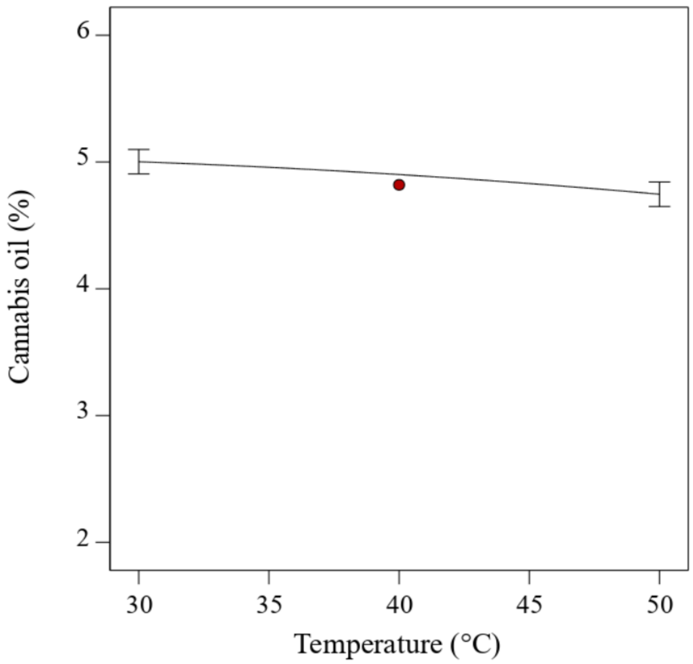

Fig. S2: Effects of temperature on cannabis oil yield at constant CO_2 pressure (250 bar) and extraction time (1.5 h)

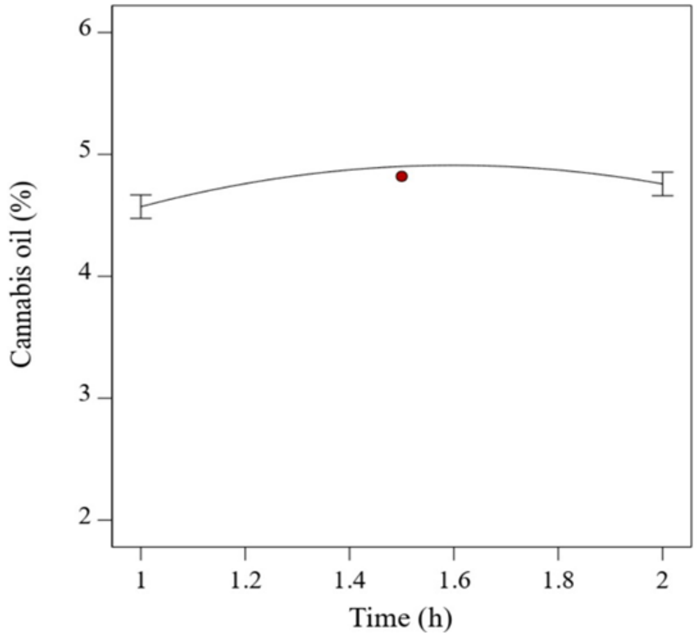

Fig. S3: Effects of extraction time on cannabis oil yield at constant temperature (40°C) and CO_2 pressure (250 bar)

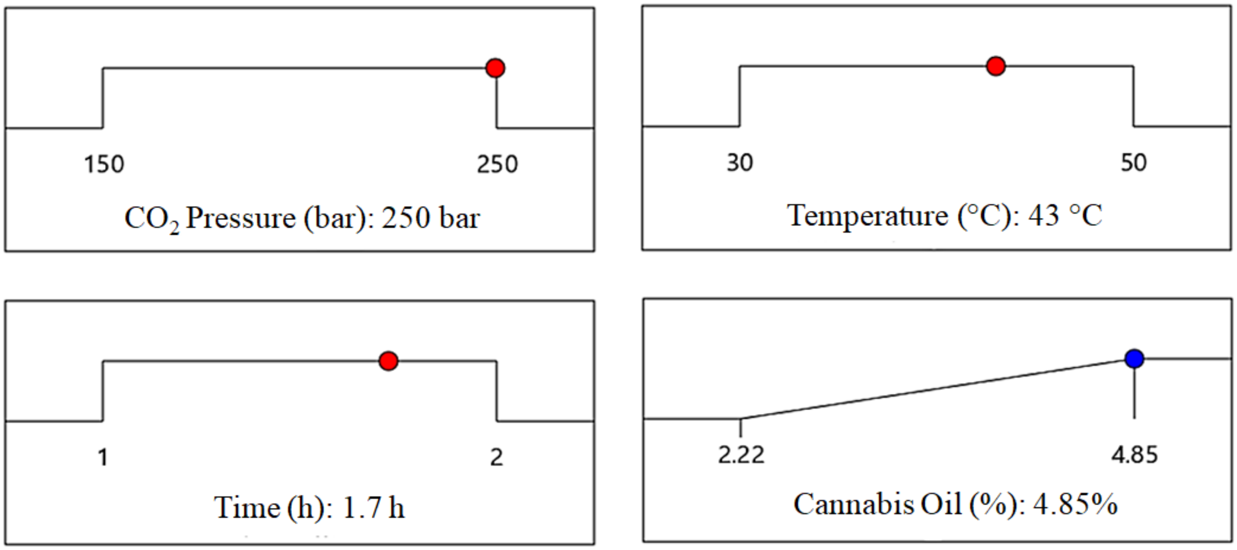

Fig. S4: Optimized conditions of $SCCO_2$ generated from the statistical model

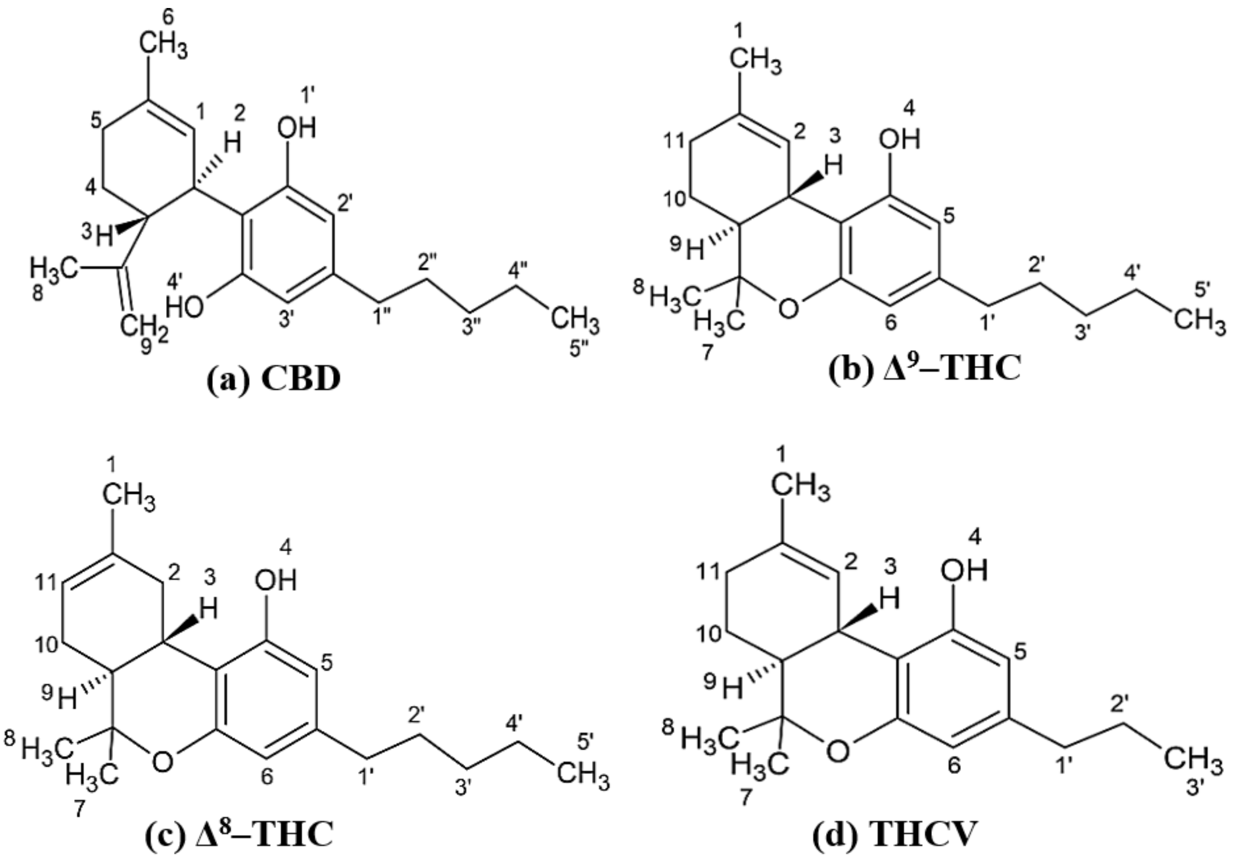

Fig. S5: Chemical structures of (a) CBD, (b) Δ^9-THC, (c) Δ^8-THC and (d) THCV.

Table S1. Comparison between the experimental and predicted yield of cannabis oil.

**Experimental run**	**Experimental value (%)**	**Predicted value (%)**	**Error (%)**
1	3.4	3.4	2.9
2	4.9	4.8	0.4
3	4.9	4.9	1.6
4	2.2	2.3	1.8
5	3.7	3.8	2.6
6	3.5	3.4	1.2
7	4.8	4.7	1.1
8	3.1	3.1	2.0
9	3.6	3.7	1.9
10	3.9	3.9	1.3
11	3.8	3.8	0.5
12	2.9	2.9	0.7
13	2.7	2.6	1.5
14	3.9	3.8	1.6
15	3.7	3.7	2.0
16	4.4	4.4	0.7
17	4.7	4.6	0.9
18	3.9	3.8	3.2

Table S2. Design of experiments for $SCCO_2$ extraction of cannabis leaves.

**Experimental run**	**CO_2 pressure (bar)**	**Temperature (°C)**	**Time (h)**
1	150	50	2
2	250	30	2
3	250	40	1.5
4	150	30	1
5	200	40	1.5
6	200	40	1
7	250	30	1
8	150	40	1.5
9	200	30	1.5
10	200	50	1.5
11	200	40	1.5
12	150	50	1
13	150	30	2
14	200	40	1.5
15	200	40	2
16	250	50	1
17	250	50	2
18	200	40	1.5

Note: The amount of biomass and CO_2 flow rate were kept constant at 100 g and 35 g/min for all the runs.
